# The feasibility and RE-AIM evaluation of the TAME health pilot study

**DOI:** 10.1186/s12966-017-0560-5

**Published:** 2017-08-14

**Authors:** Zakkoyya H Lewis, Kenneth J Ottenbacher, Steve R Fisher, Kristofer Jennings, Arleen F Brown, Maria C Swartz, Eloisa Martinez, Elizabeth J Lyons

**Affiliations:** 1 University of Texas Medical Branch, 301 University Blvd, Galveston, TX 77551 USA; 2 Beachbody LLC, 3301 Exposition Blvd, Santa Monica, CA 90404 USA; 3 University of California Los Angeles, 200 UCLA Medical Plz, Los Angeles, CA 90095 USA

**Keywords:** Physical activity, Technology, Primary care, Activity monitor, Older adults, RE-AIM, Pragmatic, Jawbone, Pedometer

## Abstract

**Background:**

Conducting 5 A’s counseling in clinic and utilizing technology-based resources are recommended to promote physical activity but little is known about how to implement such an intervention. This investigation aimed to determine the feasibility and acceptability, using the RE-AIM (Reach, Effectiveness, Adoption, Implementation, Maintenance) framework, of a pragmatic, primary care-based intervention that incorporated 5 A’s counseling and self-control through an activity monitor.

**Methods:**

Primary care patients (*n* = 40) 55–74 years of age were recruited and randomized to receive a pedometer or an electronic activity monitor (EAM), Jawbone UP24, to monitor activity for 12 weeks. Participants were also invited to a focus group after completing the intervention. Stakeholders (*n* = 36) were recruited to provide feedback.

**Results:**

The intervention recruitment rate was 24.7%. The attrition rate was 20% with a significantly higher rate for the pedometer group (*p* = 0.02). The EAM group increased their minutes of physical activity by 11.1 min/day while the pedometer maintained their activity (0.2 min/day), with no significant group difference. EAM participants liked using their monitor and would continue wearing it while the pedometer group was neutral to these statements (*p* < 0.05). Over the 12 weeks there were 490 comments and 1094 “likes” given to study peers in the corresponding application for the UP24 monitor. Some EAM participants enjoyed the social interaction feature while others were uncomfortable talking to strangers. Participants stated they would want counseling from a counselor and not their physician or a nurse. Other notable comments included incorporating multiple health behaviors, more in-person counseling with a counselor, and having a funding source for sustainability.

**Conclusions:**

Overall, the study was well-received but the results raise a number of considerations. Practitioners, counselors, and researchers should consider the following before implementing a similar intervention: 1) utilize PA counselors, 2) target multiple health behaviors, 3) form a social support group, 4) identify a funding source for sustainability, and 5) be mindful of concerns with technology.

**Trial registration:**

clinicaltrials.gov- NCT02554435. Registered 24 August 2015.

**Electronic supplementary material:**

The online version of this article (doi:10.1186/s12966-017-0560-5) contains supplementary material, which is available to authorized users.

## Background

Habitual physical activity (PA) can reduce risk for cardiovascular diseases (CVD) [1–6], but most older adults fall far below the recommended 30 min of moderate intensity PA at least 5 times a week [7–9]. There are several reasons why older adults do not get enough PA including social influences, competing priorities, personal beliefs and motivation [10]. The American Heart Association encourages the implementation of individual clinical and population-level strategies to target these barriers and reduce physical inactivity [11]. One such strategy is to implement 5 A’s counseling within the primary care clinic [11].

The 5 A’s stand for assess, advise, agree, assist, and arrange [11, 12]. Five A’s counseling was developed by the Counseling and Behavioral Interventions Work Group of the United States Preventive Services Task Force to provide brief counseling within the primary care setting [12]. This form of counseling is recommended over comprehensive counseling because it is short in duration and more feasible for a busy clinic [11, 13–17]. The assist component is particularly impactful because the clinician provides behavioral change techniques, such as problem solving and social support, that aid in changing PA behavior [18]. Shaping knowledge and providing feedback through counseling are effective behavioral strategies [19] but the addition of technology is recommended to enhance counseling for individuals at moderate risk for CVD [11].

Activity monitors have the potential to enhance primary care interventions by motivating individuals to change their PA behaviors [20] while lessening the burden on clinical staff [11, 21]. Two types of activity monitors are commonly used for PA promotion: pedometers and electronic activity monitors (EAMs). Pedometers are low-tech devices that provide immediate feedback on PA and have been shown to be feasible and acceptable within primary care interventions [22–24]. Conversely, EAMs are high-tech devices that can provide PA feedback, individualization, and behavior change techniques (e.g. action planning, social comparison, and cues to action) [20, 25]. There is preliminary evidence that these monitors are feasible in community interventions [25]. EAMs have potential in primary care because they offer effective behavior change techniques that may be overlooked in clinic-based counseling and they facilitate social support [26]. Positive social interaction, such as providing encouragement, can further promote PA because it provides emotional support [27, 28]. Furthermore, it allows older adults to learn PA tips from their peers [29] and it is associated with long-term behavior maintenance [30].

The American Heart Association recommends a 2-tiered approach to promote lifestyle changes, like PA, in the healthcare system. The first tier is to provide low-intensity 5 A’s counseling and the second tier is to utilize technology-based resources [11]. However, the adoptability of a primary care-based intervention that incorporates counseling and activity monitoring has not been studied in depth. There is also limited information on how to successfully combine and implement these types of interventions in a real world setting [31].

To increase the likelihood that research findings will be utilized in the clinic, interventions need to be pragmatic [32–35] and they should be assessed for their impact on the population-level [36]. The pragmatic nature of a study can be illustrated with the Pragmatic Explanatory Continuum Indicator Summary (PRECIS) figure. It’s recommended that the PRECIS figure should be created while designing an intervention to determine if it is explanatory or pragmatic. Population-level impact can be assessed through RE-AIM indicators [37]. RE-AIM is a public health framework that describes the reach, effectiveness, adoption, implementation, and maintenance of a program [36]. The purpose of the current study was to determine the feasibility and acceptability of the TAME health (Testing Activity Monitors’ Effect on health) pilot intervention within the RE-AIM framework using dimension indicators outlined by Harden et al. [37] TAME health is a pragmatic, primary care-based pilot intervention that incorporates 5 A’s counseling and self-control through an activity monitor. Furthermore, we aimed to compare feasibility and acceptability results between two types of activity monitors: pedometer (Digi-walker CW-700/701, YAMAX, San Antonio, TX) and EAM (UP24 by Jawbone, San Francisco, CA).

## Methods

TAME Health is a short-term pilot study. The methodology for this study has been previously described in-depth, [38] and the study is registered online at clinicaltrials.gov (NCT02554435). The methodology related to feasibility and acceptability outcomes is described briefly below.

### Recruitment

Study participants (*N* = 40) were recruited from two primary care clinics affiliated with The University of Texas Medical Branch (UTMB). Clinic patients were recruited by direct solicitation from the clinic lobby and flyers posted throughout the clinic. Patients were screened for eligibility in person or over the phone. Patients were deemed eligible if the following criteria were met: age (55–74 years), physically inactive (self-reported less than 60 min/week of planned PA), body mass index between 25 and 35, healthy enough for exercise measured by the PA Readiness Questionnaire Plus (Par-Q+) [39] and access to a smart device. After participants were deemed eligible an initial assessment was scheduled.

In an effort to assess adoptability, stakeholders (*n* = 36) were also recruited. Stakeholders were staff and faculty members of the medical institution who could provide input on clinic-based practice. They were recruited via an institutional email list to take part in a focus group meeting.

### Intervention procedures

All study participants underwent 5 A’s counseling from a counselor during their first assessment prior to randomization. The counselor was trained in exercise physiology and motivational interviewing. Counseling lasted approximately 5 to 10 min and the counselor followed a script (Additional file 1). In addition to the traditional 5 A’s, the counselor also provided an exercise prescription that summarized the goals and action plans agreed upon during counseling [40]. After counseling, participants were randomized to the pedometer group or the EAM group using a random number generator [41].

Participants in the pedometer group received the Digi-Walker CW-700/701 digital pedometer (YAMAX, San Antonio, TX). The participants also received an activity log to record their daily steps, activity time, and distance walked measured by the pedometer.

Participants randomized to the EAM group received an UP24 wearable device manufactured by Jawbone. They were instructed to install the corresponding UP application (app) to their smart device and wear the bracelet monitor daily. The UP24 was chosen for the intervention due to its popularity and the potential for interactive tools such as Jawbone’s “Smart coach” to be utilized [20, 42]. In addition to monitoring PA behavior, the UP24 also measures sleep and the app allows for participants to track their diet and weight. All participants were given an anonymous UP app account and encouraged to socialize with other participants in the group through the app.

### Assessment procedures

Feasibility was operationalized through evaluation of attrition, the number of days logged for activity, reported adverse events, report of technical difficulties, and social interactions in the UP app. Logged days of activity were taken from the pedometer log and from the Jawbone online data file. The effectiveness of the intervention to increase minutes of PA was measured with a SenseWear Armband. Although the Jawbone UP24 measures minutes of PA, the Sense Wear Armband was used as an assessment tool to ensure PA was measured the same way in both study groups. The SenseWear armband is validated to estimate steps per day and minutes of moderate to vigorous physical activity [43, 44]. Participants were instructed to wear the armband for a 7-day period at baseline and at 12 weeks. Participants were not excluded from the study if the objective measurement of PA was greater than 60 min per day. The effectiveness of the participants to self-regulate their behaviors was measured by the Exercise Goal-Setting Scale and Exercise Planning and Scheduling Scale [45]. Both scales are self-report and were administered at baseline and at 12 weeks.

Acceptability was determined through questionnaires and focus groups. Questionnaires included 16 items for the pedometer group and 37 items for the Jawbone group which allowed participants to answer on a range from 1 (strongly disagreed) to 5 (strongly agreed) for each acceptability statement. Statements were modeled on items previously developed by Vandelanotte et al. [46] There were additional questions related to the acceptability of different EAM features which resulted in more questionnaire items for the Jawbone group compared to the pedometer group (see Table 3). Focus groups were chosen over exit-interviews to allow the opportunity for EAM participants to meet their peers with whom they interacted through the app. Participant focus groups included 2–8 individuals and were based on a structured guide (Additional file 2). The discussions were led by two trained Masters-level interns who were well-versed on the study protocol and the study activity monitors. Focus groups were broken up by intervention group and clinic location.

Additional focus groups were conducted with stakeholders. These discussions were led by the principal investigator, ZHL. During the focus group, stakeholders were prompted to fill out two brief quantitative surveys. After the focus group, stakeholders had the opportunity to test the UP24 monitor for 4-weeks and provide feedback on usability.

Feasibility and acceptability results were organized into dimensions of RE-AIM [37]. Indicators of Reach included the recruitment rate, participant characteristics, and focus group participation rates. Effectiveness included follow-up results of PA and self-regulation, percent attrition, rates of adverse events, and quantitative acceptability results. Qualitative comments about the delivery of the interventions were used as indicators of Adoption. Implementation indicators included the report of technical difficulties, number of logged activity days, and participant perceptions of the intervention. Maintenance was divided into individual and organizational. Indictors of individual maintenance included quantitative and qualitative results of each monitor’s usability. Stakeholder perceptions of the intervention were used as an indicator of potential organizational maintenance.

### Statistical analyses

The Statistical Package for the Social Sciences (SPSS, version 20) and NVivo 11 Pro (QSR International) were used to perform the quantitative and qualitative analyses, respectively. The α-level was set at 0.05. Descriptive statistics across intervention groups were calculated by means, medians, and frequencies. Comparisons between groups for feasibility and acceptability were analyzed by an Independent T-Tests and Chi-Square tests. PA, exercise goals, and exercise planning was assessed with an analysis of covariance using the intent-to-treat principle (carrying baseline measurements forward) and controlled for baseline values of the dependent variable. Although exercise goals and planning were self-reported, results are presented in means and standard deviation [47]. Cohen’s d effect sizes were calculated using the change mean change in PA, exercise goals, and exercise planning.

Thematic analyses were conducted to analyze data from the focus groups [48]. We chose thematic analysis because we wanted to describe participant and stakeholder perspectives of the main study components. Initial codes were developed prior to the focus groups and new codes were added based on new data. All focus groups were audio-recorded and data transcripts were written out by the principal investigator. The moderators were asked to verify any inaudible segments.

### Pragmatic evaluation

The PRECIS-2 figure (Additional file 3) was used to illustrate the explanatory and pragmatic components of the study [33, 34]. Study components, including eligibility, recruitment, setting, organization, flexibility-delivery, flexibility adherence, follow-up, primary outcome, and primary analysis, were rated on a 1–5 scale with 5 being the most pragmatic. The figure for this study was rated by Principal Investigator and illustrated that this intervention was largely pragmatic.

## Results

Complete demographic information is illustrated in Table 1. Feasibility and acceptability results are described below by each dimension of the RE-AIM framework [36, 37]. Complete quantitative results are presented in Tables 2 and 3. Feedback from the focus groups was centered around 4 major themes: TAME health, self-monitoring, social support on the UP app, and counseling from the counselor or from a health care provider. Example quotes from the focus groups are presented in Table 4.

**Table 1:** Participant demographic information (*n* = 40)

	Electronic activity monitor (*n* = 20)	Pedometer (*n* = 20)	All (*n* = 40)
Age, years; mean (SD)	64.0 (5.1)	63.2 (5.7)	63.7 (5.3)
Body mass index, kg/m^2^; mean (SD)	30.0 (3.2)	30.6 (3.1)	30.3 (3.1)
Moderate/Vigorous physical activity minutes; mean (SD)	22.5 (21.5)	40.0 (33.9)	31.3 (29.37)
Steps per day; mean (SD)	3849.4 (2107.8)	4560.18 (22.85.6)	4204.8 (2199.8)
Female, n (%)	17 (85)	13 (65)	30 (75)
Hispanic, n (%)	3 (15)	2 (10)	5 (12.5)
Black/African American, n (%)	4 (20)	3 (15)	7 (17.5)
Other, n (%)	1 (5)	1 (5)	2 (5)
Non-Hispanic White, n (%)	12 (60)	14 (70)	26 (65)
College or Graduate/Professional school, n (%)	10 (50)	12 (60)	22 (55)
Some college or technical school, n (%)	8 (40)	7 (35)	15 (37.5)
High school diploma/General education development, n (%)	2 (10)	1 (5)	3 (7.5)

**Table 2:** Feasibility results

	Pedometer (*n* = 20)	Electronic activity monitor (*n* = 20)
Days of recorded step data, mean (SD)^b^	71.4 (11.5)	73.1 (21.5)
Attrition rates, n (%)^a^	7 (35)	1 (5)
Moderate/high adverse events, n (%)	1 (0.05)	3 (15)
Report of technical difficulties, n (%)	10 (50)	13 (65)
UP app usage		
“Likes” given through the UP app, median (IQR)		0.0 (38)
“Likes” on user’s own activity, median (IQR)		3.5 (31)
Comments given through the UP app, median (IQR)		0.0 (15)
Comments on user’s own activity, median (IQR)		0.0 (4)

*app* application, *IQR* Inter-quartile range, *SD* Standard deviation

^a^ *p* < 0.05

^b^The reported means and standard deviations are based on participants with complete step data (pedometer, *n* = 9; electronic activity monitor, *n* = 19). Pedometer step data was based on returned physical activity logs. Electronic activity monitor step data was retrieved from an online server

**Table 3:** Acceptability results from the follow-up questionnaire

	Pedometer (*n* = 12)	Electronic activity monitor (*n* = 19)	Stakeholder (*n* = 6)
Feelings about the study	Mean (SD) *Range: 1 to 5*		
I felt the counseling was motivational	3.8 (1.2)	4.1 (0.7)	
The exercise prescription was helpful	3.8 (0.8)	3.8 (1.0)	
I would prefer if there were more counseling sessions	3.0 (1.0)	3.5 (1.3)	
Feelings on the activity monitor			
It was easy to remember to wear the monitor	3.5 (1.6)	4.5 (0.8)	3.2 (1.33)
I felt that the monitor was comfortable	4.2 (1.2)	4.1 (1.0)	4.2 (1.0)
I would continue to wear the monitor^a^	3.5 (1.5)	4.4 (0.9)	3.7 (1.2)
The monitor was motivating	3.7 (1.1)	4.4 (1.0)	3.5 (0.8)
I liked using the monitor^b^	3.0 (1.4)	4.4 (1.0)	4.0 (0.6)
I thought the pedometer was helpful	3.3 (1.4)		
I would prefer to use another type of monitor	3.1 (1.3)	2.4 (1.3)	2.0 (0.9)
I have a better understanding on my physical activity level	3.8 (1.1)	4.4 (1.1)	3.5 (1.0)
Feeling on the Jawbone UP application			
It was convenient for me to use the UP application		4.6 (0.8)	4.2 (0.8)
The UP application encouraged me to view my steps		4.7 (0.7)	4.2 (0.8)
I would like to continue using the UP application		4.5 (1.1)	3.8 (0.8)
I think the application is user-friendly		4.3 (1.2)	3.8 (0.8)
I enjoyed the social interaction		3.7 (1.0)	N/A
Comments and smiles from my “friends” in the application were motivating		3.9 (1.2)	2.7 (0.8)
I think the information is interesting		4.6 (0.5)	4.0 (0.6)
I think the information is relevant		4.5 (0.6)	3.8 (0.4)
I think the tips and advice are specific to me		4.0 (0.9)	3.8 (0.8)
I am going to use the advice		4.4 (0.7)	3.3 (0.5)

^a^ *p* < 0.05, ^b^ *p* ≤ 0.01, Significantly different between pedometer and Electronic activity monitor group

**Table 4:** Example quotations from study groups

	Electronic activity monitor	Pedometer	Stakeholder
TAME health study	Q: Do you think that your attitude towards exercise has changed? *It’s a good idea. I think, I would really advise it for anybody that wants to try and get themselves going. I think belonging to something like this is a really good idea. Female, 73*	Q: Do you think that your attitude towards exercise has changed? *That was an education for me. Just to wear it and to see how much I actually did in a day’s time. Female, 72*	Q: What are your thoughts about the study? *Even though people dropped out and there was damage with some of the monitors, just the fact that every group increased their activities and their steps. I think you can definitely tell that this encouraged and motivated them to be more active than they usually would have been.*
Self-Monitoring	Q: Do you think you’ll continue using a monitor like this? *I even would like to get one for myself and my husband so we can both keep track of our activity. Female, 73*	Q: Do you think you’ll continue using a monitor like this? *I would probably go out and buy one because I think it’s really good psychologically, to have something that you can actually see. Female, 72*	Q: Would you recommend your patient to use this monitor? *If we didn’t have it to give to them, they couldn’t afford it.* Q: Do you currently advise your patients to monitor their activity? *There’s certain patients that you know are going to be engaged in that way and others that if you told them that was an additional step that would maybe turn them away from it.*
Pedometer		Q: Thinking about the monitor you used, what did you like about it? *I like the convenience of knowing how active I’ve been or how inactive that I’ve been, and what time frame. Female, 72* Q: What didn’t you like about it? *It also irritated my belly, you know that’s where I had it sitting. Female, 66* Q: What didn’t you like about it? *You had to have it in a certain position on your waist or it wouldn’t read. I had a week where it didn’t read for a few days. Male, 56*	Q: Would you recommend your patients to use this pedometer? *The belt clip ones, well relatively, they’re the ones that are bound to go in the toilet or dropped.*
UP24	Q: Thinking about the monitor you used, what did you like about it? *I liked the Jawbone because it was comfortable. I would put it on in the morning and I never felt it again. It didn’t bother me at all, and it’s not awful looking. Female, 55*		Q: Would you recommend your patients use this monitor? *I think that recommending this to someone who is comfortable with technology. Otherwise, if you go bombard them with all these types of data and statistics, they may not know exactly what it is they they’re looking at.*
	Q: What didn’t you like about it? *No complaints except that they’re going out of business. That’s why I didn’t buy one. Female, 68* Q: What didn’t you like about it? *My neighbor was in the program also, ahead of me… She was energized and she had the iPad, which is more conducive or complimentary with it. I have an [Android] tablet. So the interface for me was not as gratifying as hers. It worked again me which I didn’t need. Female, 68*		Q: Would you recommend your patients use this monitor? *I would think something like this for most people in this room works. We all have smartphones, but in terms of the patients we see. I would say maybe one out of every 10 have a smartphone. A good portion of them don’t have Internet at home so connecting to an app or something like this is pretty… even though it’s prettier and great for us it may not be useful for an older population.*
Social support on UP app	Q: How often did you communicate with other participants? *I was on there maybe a week when I met cowboy. She kept hitting goal and she had a small goal because she [had] knee surgery and I said ‘do you want to meet to walk?’ I just assumed everybody was in Galveston.* *Well she wasn’t, she was in League City. So we met in Texas City. Female, 64* Q: How often did you communicate with other participants? *If I saw that somebody had done a lot that day I would give them a thumbs up and stuff like that… and then other people would encourage me and I didn’t know who they were either but their icon. Female, 68* Q: How often did you communicate with other participants? *I understand the rationale for the anonymity but I’m just sitting here thinking now, that for me, probably if I met everybody I would have been much more social. Female, 61*		Q: Would you recommend your patients use this monitor? *Even for me as an adult. I have friends that we do like that challenges that you can do. Even as adults that’s something we do very regularly. So see who can win.*
Counseling from the counselor	Q: Did you like receiving counseling from a counselor? *I think that’s good to have somebody else that maybe is a little more informed than your family or your friends or even reading about it. It is good just to have a face to face. Female, 73*	Q: Did you like receiving counseling from a counselor? *[I felt] very, very comfortable. It was not like that she was speaking above my head and I’m going ‘I don’t have any idea what she’s talking about’… like I said, she was always very supportive and I think that made the big difference too. Female, 72*	
Counseling from health care provider	Q: Would you prefer the counseling done by your primary care physician? *No. I’ve got a great primary care physician here but I love that it was separate from that. Ya know, because that’s my medical and this is my health. Female, 61* Q: Would you prefer the counseling done by your primary care physician? *No, I don’t think I would especially like that because I don’t think the primary care physician has time enough. They’re usually trying to take care of whatever your current problem is. They want to hit on several different things and I don’t think exercise or diet is big in their specialty area. They just either don’t take the time or don’t know enough about it to individualize it for you. So I think it’s better to have someone else do that. Female, 73*	Q: Would you prefer the counseling done by a nurse, physician assistant, or anyone else who worked with your doctor? *It’s just like those two things seem to be separate in my mind. I just feel like they, everybody in the doctor’s office is so busy. With [the counselor], I felt that she had all the time in the world to deal with me. Female, 66* Q: Would you prefer the counseling done by your primary care physician? *[My physician] takes care of my booboos and my ouches and ooze and ‘what-the-heckes’ kind of stuff. This other thing… I mean, they’ll sit and tell you ‘you need to exercise Hunny’ but they don’t’ really give you a plan or that’s just not their job. Female, 72* Q: Would you prefer the counseling done by a nurse, physician assistant, or anyone else who worked with your doctor? *In my opinion a nurse or a doctor, they’re not… they’re a doctor. They can give you some advice on how to… what you need to do but as far as how to get it done, that’s not their area of study. Male, 5* *6*	Q: Would you use this counseling with your patients? *It just depends…if there was a part of the process that said, hey the social worker can go in and do the counseling for this [patient] versus taking away a MA or a nurse that would be maybe triaging or drawing blood or doing something else.* Q: Would you use this counseling with your patients? *Would I tell my nurse to do that? Yes. Or would I ask my nurse to do that and would she participate in that, yes. I mean, I think I do have support staff that will do that. Do I think it’s important? Absolutely.*
5A’s structure	Q: Of the counseling components, which did you value the most? *You have to advise with assess. Without… looking at those numbers probably would not have meant as much. Female, 64*	Q: Of the counseling components, which did you value the most? *Maybe number 2 [advise]… If I was going to do this, I was going to do what I was told to do. Female, 72*	Q: Have you done 5 A’s counseling in your clinic? *I’ve actually done it without knowing it. It’s pretty much what I do with patients.*
	Q: Of the counseling components, which did you value the most? *I think with [the counselor] suggesting, well maybe you can start with at least 5000 [steps], it gave me something to work toward. So I eventually did get there and I even got up to where it was not unusual for me to get 10,000 steps. Female, 61*	Q: Of the counseling components, which did you value the most? *Agree, reach agreement, that helped too because you can set a goal then. Male, 56*	
Exercise prescription	Q: Did you find the written prescription helpful? *It was [helpful]. I have a hard time remembering things and forming habits so this was up on my board. So I saw it every day. I would say ‘Oh that’s right my goal’s 7000’ what do I have, oh I only have 4 [thousand], I better go for a walk. That helped, but it was a starting point and it was necessary. Female, 64*	Q: Did you find the written prescription helpful? *Everything I got was really [an] eye opener because of seeing it in writing*, *and it’s just to you. It’s not the household or anything.* *It’s only up to you to do these things that are listed here. So that was a* *good prescription*. *A good incentive to look and see. Female, 72*	Q: Do you like the counseling? *To me it seems the prescription was almost more motivating in the sense that now they have something they received from a clinician that says, this is what I recommend to you*.

### Reach

**Fig. 1:**
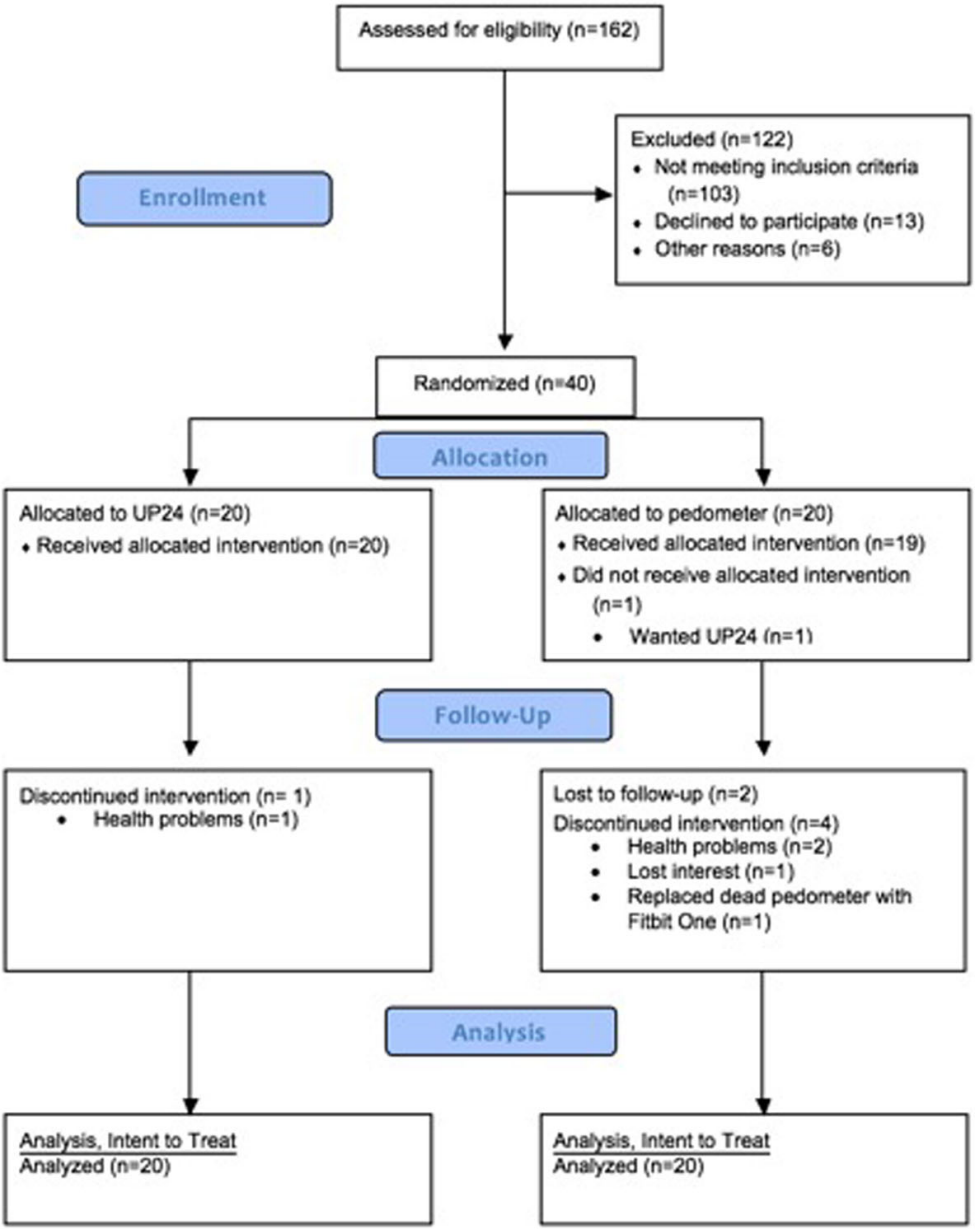
Recruitment flow diagram

Recruitment rate is displayed in Fig. 1. A total of 162 individuals were screened for eligibility over 8 months (October 2015–June 2016), and 42 were eligible. Two eligible participants dropped out before randomization due to care-giver responsibilities and work commitments. The resulting recruitment rate was 24.7%. Twenty-seven (67.5%) participants were recruited in-person at the clinic, 8 (20%) were recruited through flier postings, and 5 (12.5%) were referred by a friend or employee that heard of the study from the clinic. Eight (20%) participants were not clinic patients. Four of these individuals were referrals while 4 were recruited in-person while at the clinic with their family.

At baseline, participants had a mean age of 63.7 ± 5.3 years (EAM: 64.0 ± 5.1, Pedometer: 63.2 ± 5.7). Most participants were female (total: 75%, EAM: 85%, Pedometer: 65%), non-Hispanic White (total: 65%, EAM: 60%, Pedometer: 70%), and had a college degree (total: 55%, EAM: 50%, Pedometer: 60%). The EAM group and the pedometer group averaged 22.6 ± 24.5 and 40.0 ± 33.9 min of moderate or vigorous PA a day, respectfully. There were no significant group differences among these variables. Stakeholders were predominately clinical faculty and professors (33%). Other stakeholder positions included physician, nurse, social worker, graduate student, epidemiologist, researcher, research coordinator, post-doctoral fellow, and administrator.

There were 6 scheduled focus group meetings for study participants. Of the 36 study participants that were invited to take part in a focus group, 11 participated (8 EAM, 3 Pedometer). Four participants were not invited because they ended the intervention several weeks after the majority of participants. Although focus groups were planned, several of the meetings (4 out of 6) resulted in a one-on-one interview due to low attendance. These interviews followed the same structured question guide. There were two scheduled stakeholder focus group meetings that reached 36 individuals.

### Effectiveness

Over 12 weeks the EAM and pedometer group increased their minutes of moderate or vigorous PA by 11.1 and 0.2 min per day, respectively. The groups were not statistically different in their rate of PA at 12 weeks (*p* = 0.29, d = 0.78). Groups were significantly different in exercise goal-setting and planning scales (*p* < 0.01). The EAM group increased 8.3 ± 9.8 and 3.6 ± 7.6 points in goal-setting and planning while the pedometer group increased by 1.7 ± 5.3 and 0.3 ± 4.1 points, respectively. The resulting effect size was large for goal-setting (d = 0.84) and planning (d = 0.55).

Overall attrition was 20%, which differed significantly between groups (*p* = 0.02). Two participants (pedometer group) were lost to follow up and 6 participants (EAM: 1, Pedometer: 5) did not finish the intervention. The EAM participant dropped out due to physical health issues. Reasons for pedometer participants not completing the intervention included: randomized to the pedometer but wanted the EAM (*n* = 1), physical health issues (*n* = 2), lost interest (*n* = 1), and replaced broken pedometer with an EAM (*n* = 1). Participants that dropped out had a significantly higher goal-setting score at baseline. In addition to the drop-outs, two participants (EAM: 1, Pedometer: 1) did not complete the final assessment but provided PA data and/or subjective data.

There were no moderate or severe adverse events related to the study. However, there were four moderate unrelated adverse events during the study.

Only 7 stakeholders agreed to wear the UP 24 and all but 1 provided feedback. Of these, 3 only tested the monitor and did not take part in a focus group. Study participants and stakeholders agreed that the study and UP24 monitor were mostly acceptable. EAM users agreed that they liked using the monitor and that they would continue wearing it while pedometer users were neutral to these statements (*p* < 0.05).

### Adoption

Participants felt that the doctor’s office was too regimented, and this study is something they did for themselves, not for their doctor. Moreover, the participants expressed that PA is separate from primary care. As one participant stated “I’ve got a great primary care physician here but I love that it was separate from that… because that’s my medical and this is my health (Female, 61).” The study participants enjoyed counseling from the counselor and would not want counseling from a health care provider. However, they would like if the counselor was part of the health care team and had access to their medical record. Participants wanted more in-person counseling sessions and counseling on other health behaviors. The EAM group found all of the 5A’s components helpful while the pedometer group found “Advise” the most helpful. The exercise prescription after counseling was also helpful to participants.

Stakeholders liked the counseling format but stressed it would need to be individualized to the patient. Practitioners often use one of the 5A’s components but do not recognize it as 5A’s counseling. Stakeholders also commented that the physician would not have time to conduct the “arrange” call but it could be done by a clinical staff member. Like study participants, stakeholders liked the exercise prescription. They would alter the prescription to have more “I will…” language, more planning details, and prescribe both steps per day and minutes of PA.

### Implementation

There were 28 reports of technical issues across 21 participants during the intervention. All broken or lost monitors were replaced and all other technical issues were resolved. Five pedometers were lost, 5 pedometers broke, and participants sought help from the research staff for assistance with getting data from the pedometer on 3 occasions. There were 2 reports that the UP24 would not hold a charge, 5 reports of UP24 Bluetooth connectivity issues, 3 reports that the UP24 would not record activity and 1 UP24 was lost. During the intervention 4 participants got a new phone or downloaded the UP app on a different device. Three of these participants sought assistance from the research staff while 1 participant created their own UP account and was no longer connected to the rest of the group. Over the 12 weeks participants logged an average of 72.6 days of activity. There was no difference between groups in logging at least 80% of activity days.

Overall participants reported liking the TAME health program. Participants found it educational to know how active they were in a day and the monitor they used (pedometer or EAM) became a part of them. One of the most motivational aspects that participants reported was having a goal in mind. Despite enjoying the program, participants wanted a multiple behavior change intervention that also targeted water consumption, diet, and sleep.

### Maintenance: individual

Both monitors were admired for being easy to use, convenient, and discreet. Of the participants that used the UP app, some liked that it synced with another device while others wanted direct feedback on the monitor. The app was reported by most to be user friendly. Participants in both study groups questioned the accuracy of the devices and how they recorded the activity. Both groups also complained that the device could irritate the skin. EAM users disliked the number of technical and syncing issues, as well as the interface on Android versus Apple products. Furthermore, EAM users were confused by some of the biometrics presented in the UP app. In particular, the counselor explained the concept of active and resting energy expenditure during the assess portion of counseling. Yet, participants didn’t understand how “resting burn” (estimated resting energy expenditure) could be higher than “active burn” (estimated active energy expenditure). Pedometer users disliked that the pedometer would only count steps when worn in a certain position. In the face of complications, participants would continue to use the type of device they wore.

**Fig. 2:**
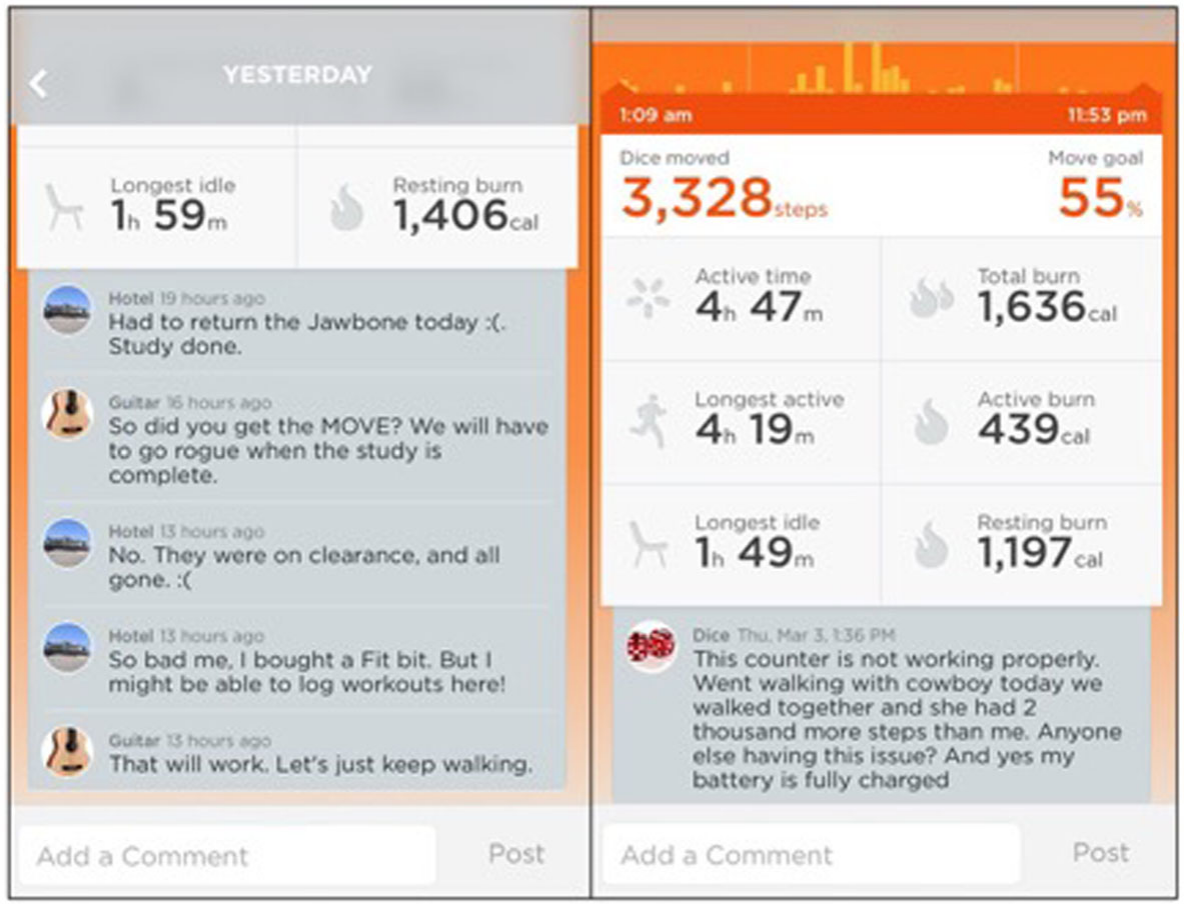
Social Interaction on the UP app

Over the 12 week intervention there were 490 comments to study peers and 299 self-comment (comments on the user’s own activity) on the UP app. There were 1094 “likes” given and 104 “likes” on the user’s own activity. Ten participants did not give any “likes” or comments to their peers and only 3 of these 10 had at least 1 self-comment. Despite this, every participant received at least 1 comment and 1 like from one of their peers. The most comments and “likes” given by a single participant was 315 and 434, respectively. During the 12 weeks, participants had 10 to 19 peers to interact with. Comment examples are presented in Fig. 2. Some EAM participants reported enjoying the socializing features and used them regularly. Others did not use any of the social support features, reporting that they did not know the other participants or their health status.

Stakeholders believed that self-monitoring may be very beneficial for some patients but not others. They were concerned with the cost of the monitors and their accuracy. For pedometers, stakeholders felt that it may work with an older population but pedometers have limitations. Some limitations cited by the stakeholders include: flimsy, bulky, sensitive to measurement, difficult to read, short battery life, and easy to lose. Stakeholders felt that the EAM is carefree, easy to wear, and it has some attractive features (i.e. competition, Smartcoach). However, there may be a technology barrier for use by patients.

### Maintenance: organizational

Prior to the focus group presentation, 90.6% of stakeholders believed that counseling is effective to change behavior while 53.1% actually counselled patients on becoming more physically active. Similarly, 84.8% of stakeholders believed that activity monitors can change behavior while 18.8% advised patients to use an activity monitor. After the presentation, stakeholders somewhat agreed that they would recommend an EAM over a pedometer for their patients (3.4 ± 1.2 out of 5) and that the intervention can be implemented into their clinic (3.3 ± 1.1 out of 5). Despite this, stakeholders had positive reactions to TAME health. They felt that counseling is already a part of practice behavior and the other aspects of the study can be implemented into the clinic if there was a funding source, like grants or insurance, to supply patients with monitors.

## Discussion

The purpose of this pilot study was to evaluate the feasibility and the acceptability of a pragmatic, primary care-based PA intervention within the context of the RE-AIM framework. Overall, the study was feasible with adequate retention, sufficient number of days of recorded activity, and no study-related adverse events. The study was reasonably acceptable for participants and stakeholders. Notable comments include incorporating multiple health behaviors, more in-person counseling with a counselor (not a health care provider), and having a funding source to supply activity monitors to patients. Based on the feasibility and acceptability scores, the EAM intervention appears to be more feasible and acceptable than the pedometer intervention on some indicators of reach, effectiveness, implementation, and maintenance.

Our reach, effectiveness, and implementation findings are comparable to other primary care-based studies and interventions that utilized an EAM. Our retention rate of 80% is within the 60.7 to 95% retention rate cited in other primary care-based studies [23, 49, 50] and an EAM yields a lower attrition rate than health education alone [51]. Similar to a Fitbit-based intervention [52], we saw no adverse events related to the intervention. Only 1% of our participants had an unrelated event which is lower than the 2–19% reported in primary care-based studies [22, 50]. Like participants in other studies [52–58], EAM participants in the present study met the 80% recommended wear time. Reports of technical issues using activity monitors vary widely from 16% [52] to 90% [57]. Approximately 50% of our participants reported an issue which is line with the 58% of chronically-ill patients that used an EAM system [31].

In terms of individual maintenance, there was less social interaction among TAME health participants compared to a previous evaluation of 35 community-dwelling adults aged 55 to 79 years using the UP app (under review). Over a 12-week intervention, the 35 participants produced 1759 comments and 3153 “likes”. With the most “likes” and comments given by one participant 986 and 344, respectively. In this evaluation, 31 out of 35 participants socialized with the app. In the present study, half of the participants that used the UP app did not give support to other participants but social interaction was still prevalent (490 comments and 1094 likes). Both the current study and previous investigation of adults 55 years of age and older found that older adults organically produce over 400 comments in 12 weeks, which is more than the reported 259 comments from college-aged adults over 12 weeks [59]. These investigations also suggest that participants naturally provide emotional support to their peers through “likes”, which are viewed as a virtual empathy tool [26].

Other indicators of potential individual maintenance in our TAME health study were similar to other activity monitor interventions. We found that participants found the EAM more helpful and participants were more likely to purchase a similar EAM. Based on previous investigations, evidence suggests that older participants find a Fitbit EAM three times more helpful than a pedometer [52], they would continue to use an EAM [60], and they would purchase an EAM over a pedometer [61]. Our participants also had similar sentiments in that the monitor made them more aware of their activity, the pedometer was enjoyable because it was simple, the EAM is easy to use and put on but can cause some irritation [57, 61].

TAME health participants and stakeholders expressed opinions related to adoption and organizational maintenance that reflect known barriers and considerations of behavioral counseling in primary care. It is suggested that counseling include multiple sessions and targets multiple health behaviors [13, 32, 62]. Patients find advising helpful but they also value all constructs of 5A’s counseling which are not often performed by practitioners [63]. There is some evidence that practitioners perceive self-monitoring effective to change behavior and easier than counseling [64]. Health care providers lack the time and skills necessarily to complete effective PA counseling [65, 66].

### Considerations for implementation

Our results raise a number of considerations. PA counseling in primary care is incentivized under Patient Protection and the Affordable Care Act and obesity counseling is covered, with stipulations, under The Centers for Medicare and Medicaid Services [65]. However, TAME health participants agreed that they would prefer counseling from a counselor over their primary care physician. The first consideration is to incorporate PA counselors in primary care. As we observed, recruiting and identifying individuals in the primary care clinic provides great reach to patients and caregivers alike and, therefore, primary care should continue to act as a platform to initiate behavioral counseling [18, 21, 67, 68]. Use of designated PA counselors in primary care is feasible and has been shown to produce favorable changes in body fat and PA [69]. Behavioral health providers are already members of the primary care team and consult with medical providers but they are underutilized for health behavior change [70]. Alternatively, community health workers (e.g. allied health professionals) could undergo specialized training and be included as part of the routine primary care practice to promote PA [11, 71]. Moreover, targeting multiple health behaviors in counseling should be considered [13, 32, 62].

Practitioners, counselors, and researchers should also consider forming a support group where patients can meet. Social support is associated with PA maintenance among older adults [72] but some individuals may be apprehensive of virtual support [29]. Our participants expressed that if they met with fellow participants, they would have socialized in the app and would not feel hesitation.

Identifying a funding source that provides monitors and technical support to patients to sustain the intervention should also be considered. We found that some individuals are willing to buy their own monitor but a funding source may still be necessary to supply technical support. Similarly, practitioners, counselors, and researchers should be conscious of potential concerns using technology. Ease of use and offering a variety to patients should be considered in selecting technologies. Further, users must be mindful of the longevity of available technologies. The SenseWear armband and the Jawbone UP24 used in this study are no longer manufactured for commercial use. Other comparable monitors are available (e.g. Fitbit, Withings, Misfit) but the type should be based on available resources and patient needs.

### Strengths and limitations

The major strength of this study is that it assessed the feasibility and acceptability of a recommended intervention to prevent CVD. It was also a comparative evaluation of two common types of activity monitors that uses a mixed-methods approach. Furthermore, we presented the pragmatic nature of the intervention and presented the results within the RE-AIM framework which directly provide a foundation for optimizing future intervention implementation and adoption.

This study is limited to the reports from participants that completed the study. With one exception, there is no acceptability information from participants who dropped out or were lost to follow up. Furthermore, there was low reporting for qualitative results and we cannot draw conclusions for participants that completed the study but did not provide feedback. Based on inclusion criteria and recruitment strategy, the results are also not generalizable to all patients and potential stakeholders, including patients that do not have access to a smart device. Furthermore, the results may not be generalizable to sedentary older adults as intended. Our sample was recruited based on self-reported PA but objective assessment from the Sense Wear found that the sample as a whole averaged 30 min of PA a day. Lastly, this is a short-term pilot study that was not able to objectively assess maintenance and should not be taken to indicate efficacy.

## Conclusion

The TAME health pilot study used the RE-AIM framework to evaluate the feasibility and acceptability of a pragmatic, primary care-based, PA intervention that incorporated 5 A’s counseling and activity monitoring. Overall, the study was well-received but the Jawbone UP24 appears to be more feasible and acceptable in some respects than a pedometer. Practitioners, counselors, and researchers should consider the following before implementing a similar intervention: 1) utilize PA counselors, 2) target multiple health behaviors, 3) form a social support group, 4) identify a funding source for sustainability, and 5) be mindful of concerns with technology.

## Additional files

Additional file 1: Five A’s Counseling Script. (DOCX 27 kb)

Additional file 2: Focus Group Guide. (DOCX 11 kb)

Additional file 3: PRECIS-2 Figure. (DOCX 221 kb)

## Abbreviations

CVD: Cardiovascular disease
EAM: Electronic activity monitors
PA: Physical activity
