# Supplementary material for: The feasibility and RE-AIM evaluation of the TAME health pilot study
Source: Int J Behav Nutr Phys Act. 2017 Aug 14;14:106. doi: 10.1186/s12966-017-0560-5 (PMC5556663; doi:10.1186/s12966-017-0560-5)
Supplement: Supplementary file 1 — Five A’s Counseling Script. (DOCX 27 kb) [file 12966_2017_560_MOESM1_ESM.docx]

Appendix 1: 5 A-S counseling guide
