# Supplementary material for: The feasibility and RE-AIM evaluation of the TAME health pilot study
Source: Int J Behav Nutr Phys Act. 2017 Aug 14;14:106. doi: 10.1186/s12966-017-0560-5 (PMC5556663; doi:10.1186/s12966-017-0560-5)
Supplement: Supplementary file 2 — Focus Group Guide. (DOCX 11 kb) [file 12966_2017_560_MOESM2_ESM.docx]

Appendix 3: Focus group guide

**PARTICIPANT FOCUS GROUP/INTERVIEW GUIDE**

Before you started the program, how did you feel about exercise?

What prevented you from getting as much exercise as you wanted?

Do you think that your attitude towards exercise has changed?

Thinking about the monitor you used, what did you like about it?

What didn’t you like about it?

Do you think you’ll continue using a monitor like this (be honest!)?

Do you think you’ll use any activity monitor after the study is over (be honest!)?

Provide a copy of the brief counseling sheet.

Of the counseling components (assess, advise, agree, assist, arrange), which did you value the most?

Did you like receiving counseling from a counselor? Why or why not?

Would you prefer the counseling done by your primary care physician? Why or why not?

Would you prefer the counseling done by a nurse, physician assistant, or anyone else who worked with your doctor? Why or why not?

Provide a copy of the exercise prescription.

Did you find the written prescription helpful? Why or why not?

For the intervention group only.

Did you like the social aspects of this study? For example: the leaderboard, the comments, smileys, and duels. How often did you communicate with other participants?

Do you think you’d use some of these social tools with your friends and family?

**STAKEHOLDER FOCUS GROUP GUIDE**

The 5 A’s counseling is brief counseling that has been adopted by the United Stated Preventive Services Task Force to promote behavioral change during a doctor’s visit.

Have you done 5 A’s counseling in your clinic?

Here is a copy of the counseling done in our study along with a supplementary exercise prescription. Please look it over.

Do you like the counseling? Why or why not?

Would you use this counseling with your patients?

Using technology to self-monitor PA is an effective method to change behavior.

Do you currently advise your patients to monitor their activity?

We gave half of our participants this pedometer to self-monitor behavior.

Would you recommend your patients to use this pedometer? Why or why not?

The other half of our participants were given this Up24 to self-monitor behavior. This monitor pairs with an app on a smart device to give feedback on behavior. This is an example of the feedback it provides.

Would you recommend your patients to use this monitor? Why or why not?

In our study we compared the effectiveness of the two devices. *Present preliminary results*.

What are your thoughts about the study?

Is this study something you can implement in your clinic?

What would you change about the study?
