# Supplementary figures and images for: The feasibility and RE-AIM evaluation of the TAME health pilot study
Source: Int J Behav Nutr Phys Act. 2017 Aug 14;14:106. doi: 10.1186/s12966-017-0560-5 (PMC5556663; doi:10.1186/s12966-017-0560-5)

Appendix 3: PRECIS-2 Figure


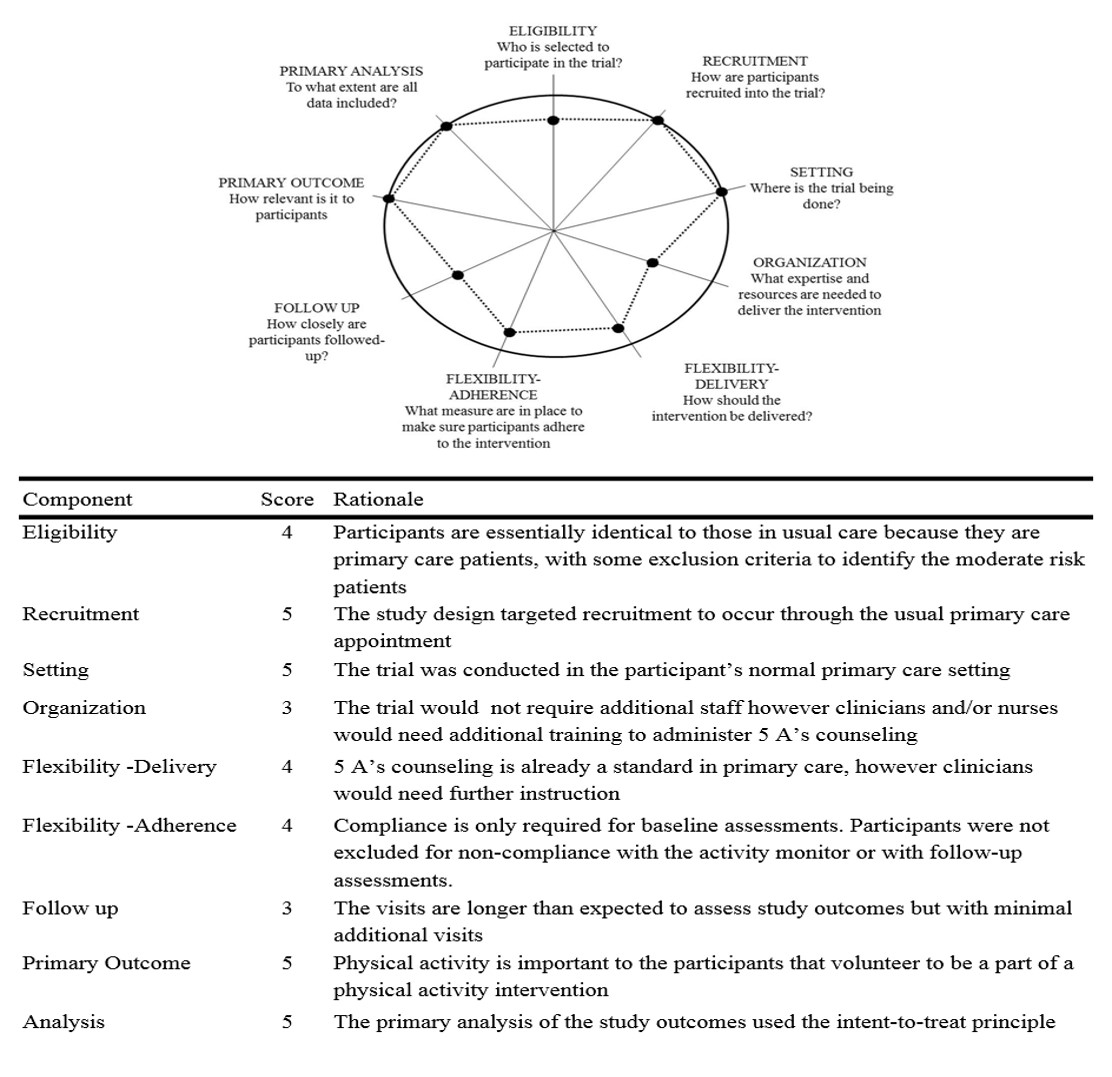

Supplement: Supplementary file 3 — PRECIS-2 Figure. (DOCX 221 kb) [file 12966_2017_560_MOESM3_ESM.docx]
